# Single-Blind Placebo-Controlled Response Test with Phenytoin 10% Cream in Neuropathic Pain Patients

**DOI:** 10.3390/ph11040122

**Published:** 2018-11-12

**Authors:** David J. Kopsky, Jan M. Keppel Hesselink

**Affiliations:** 1 Institute for Neuropathic Pain, Vespuccistraat 64-III, 1056 SN Amsterdam, The Netherlands; 2 Institute for Neuropathic Pain, Spoorlaan 2a, 3735 MV Bosch en Duin, The Netherlands; jan@neuropathie.nu

**Keywords:** topical, phenytoin, analgesia, neuropathic pain, response test, single-blind

## Abstract

Background: Phenytoin cream applied topically has been explored in neuropathic pain conditions. In several case series, phenytoin 5% and 10% cream could reduce pain in a clinically relevant way with a fast onset of action within 30 min, and with positive effects on sleep. Objective: To evaluate a single-blind placebo-controlled response test (SIBRET) for use in clinical practice. Materials and Methods: Patients with localized neuropathic pain, having an equal pain intensity in at least 2 areas (e.g., both feet), and a pain intensity of at least 4 on the 11-point numerical rating scale (NRS), were selected to perform the SIBRET. In one area, placebo cream consisting of the base cream was applied, and on the other area, phenytoin 10% cream was applied with separate hands to avoid contamination. Responders were defined as patients who experienced within 30 min at least 2-points difference as scored on the NRS, between the phenytoin 10% and the placebo cream applied areas, in favor of the former. Responders were subsequently prescribed phenytoin 10% cream. Results: Of the 21 patients, 15 patients (71.45%) were classified as responders. The mean pain reduction after 30 min as measured with the NRS in the phenytoin 10% cream area was 3.3 (SD: 1.3) and in the placebo cream area 1.2 (SD: 1.1). The difference of the mean percentage pain reduction between phenytoin 10% cream and placebo cream was 33.2% (SD: 17.6, *p* < 0.001). Using a 50% reduction on the NRS as a full response criterion, we could identify 57.1% of responders on phenytoin 10% cream and only 9.5% responders on placebo cream. Conclusions: The SIBRET helps patients and clinicians to quickly identify the appropriate treatment and can thus be seen as an important contributor to the domain of personalized medicine in pain. These results can also be regarded as a proof of principle for the analgesic activity of 10% phenytoin cream.

## 1. Introduction

Localized neuropathic pain is difficult to treat, with existing treatments requiring high numbers (between 3.7 and 9.0), and therefore, new treatment modalities are needed [1]. Topical analgesics are increasingly mentioned as putative new therapies, and phenytoin is currently one such topical active analgesic compound, together with gabapentin and baclofen [2]. In The Netherlands, analgesic creams are compounded by a local pharmacist. The challenge of such compounding analgesics is to develop an optimal base with the ability of containing high concentrations of active compounds for topical and local use, while minimizing what reaches the blood stream. An important factor for using topical analgesics safely is to inform patients what the maximum daily dose is. Furthermore, it is important to let patients know to always store topical analgesics out of the range of children and animals, making oral intake impossible.

Since 2010 we have prescribed many compounded creams, including phenytoin in a dose-range of 5% up to 30% at our Institute for Neuropathic Pain. So far, after treating more than 100 patients with phenytoin cream, there have not been reported any dose-limiting side-effects or major tolerability problems. In two cases, mild transient irritation of the skin was reported.

The effects of topical phenytoin cream in neuropathic pain has been described as a reduction of overall pain, a reduction of allodynia, and an improvement in quality of life due to positive effects on sleep [3,4,5,6,7]. Furthermore, the action of onset of clinical relevant pain reduction is generally fast, between 2 min and 30 min [8]. Its mechanism of action most probably is intradermally [9]. By applying phenytoin cream on the skin, various peripheral targets can be reached, such as the nociceptors, keratinocytes and immune-competent cells, and down-regulation of inflammatory molecules, receptors and peripheral input to the central nervous system seems possible [10,11].

The fast onset of action induced by phenytoin cream led us to develop a single-blind placebo-controlled response test (SIBRET) to allow for assessment of which part of the perceived analgesic effect can be attributed to the active compound in the cream, and which part to the placebo. Furthermore, the test helped us to identify the responders during their first clinic visit and reduce the chances that patients would, after an initial placebo response of some weeks, end up as non-responders to the cream. This SIBRET helps the patients and clinicians to rapidly identify the appropriate treatment and can be seen as one aspect in the domain of personalized medicine. Recently, in a cohort of 70 patients, SIBRET was performed in 12 patients [12]. In this article, we have also added patients who were lost to follow-up. Furthermore, the SIBRET is simplified. A responder is now defined as ≥2 points difference on the numerical rating scale (NRS) in favor of phenytoin 10% cream instead of ≥2 points pain reduction from baseline on the NRS in the active cream applied area, and a ≥1-point difference on the NRS between active cream and placebo cream area. The SIBRET, defined as a specific pharmacological challenge, is in accordance with the recently published IMMPACT (Initiative on Methods, Measurement, and Pain Assessment in Clinical Trials) recommendations on phenotyping patients in clinical trials [13].

### Single-Blind Response Test Paradigm as an Enrichment Strategy

The described single-blind test paradigm is simple and takes only a few minutes to conduct. Patients are entered in the test if they qualify as patients suffering from neuropathic pain with at least an NRS of 4. The pain has to be in two different areas and has to be preferably symmetrical, usually manifesting in both feet. The SIBRET can also be performed when one area is large enough to divide the area in 2 places (e.g., post-herpetic neuralgia, meralgia paresthetica).

One-point difference in baseline NRS between both pain areas should be the limit of accepting the patient to enter the SIBRET. If, after the SIBRET, phenytoin was clearly superior to the placebo cream, we prescribed phenytoin cream 10%. In the case of phenytoin non-responders, we would prescribe a different compounded cream, such as amitriptyline 10% cream. All creams, phenytoin, placebo and amitriptyline cream were compounded with the same base cream, and creams could not be distinguished from each other.

Our responder definition of at least 2-points difference on the NRS in pain reduction between the phenytoin and placebo cream is in line with the recommendation that a clinical relevant cut-off point for the absolute reduction on the NRS should be at least 2 [14]. Such responding patients are subsequently selected to receive a prescription for phenytoin 10% cream.

This SIBRET is a prelude to a subsequent treatment phase in responders and can therefore be seen as a way to enrich the population we treat. The term enrichment is defined by an U.S. Food & Drug Administration Guidance for Industry document as the prospective use of any patient characteristic to select a study population in which detection of a drug effect (if one is in fact present) is more likely to occur, than it would be in an unselected population [15]. The use of the SIBRET in clinical trials leads to the enrichment of the study population. Such clinical trial enrichment permits the inclusion of fewer patients, as the effect size increases.

Recently, enrichment designs have been developed for patients who are screened and selected by their biomarker status in such a way that only biomarker-positive patients enter the trial [16]. The advantage of our SIBRET compared to the biomarker approach is that the selection in our case is directly linked to the main clinical outcome parameter, pain reduction as measured on the NRS, while a biomarker is a secondary parameter only.

The question whether such SIBRET, used as a diagnostic tool to identify responders, is ethically acceptable without agreement from an institutional review board, is addressed by the American Medical Association Council on Ethical and Judicial Affairs [17]. Physicians may indeed use placebos for diagnosis or treatment, without the need to seek specific consent before administration, if patients are fully informed and agree to its use.

## 2. Materials and Methods

From February 2017 to October 2017, data was collected from patients who underwent a SIBRET in our center. Pain intensity before and after test application was measured on the NRS. One-point difference in baseline NRS between both pain areas was the limit of accepting the patient to enter the SIBRET. Furthermore, we included patients with a pain intensity of at least 4 on the NRS. Patients then received an equal amount (fingertip unit, approximately 0.6 g) of placebo cream and phenytoin cream for application on the 2 different areas, rubbing the creams in where it hurts with separate hands to avoid contamination. Responders were defined as patients who experienced within 30 min at least 2-points difference as scored on the NRS between the phenytoin 10% and placebo cream applied areas in favor of the former. The instruction given by the physician before applying the creams was: “I would like to offer you to test one cream on one pain area, which I believe can help to lessen your suffering without knowing how it exactly works. The other cream I would like to offer you to test for the other area, the working mechanism is clearer, though side effects can occur. After 30 min, you will tell us whether there is a difference in pain scoring on the NRS, and based on your evaluation we know what best to prescribe you.” This way of informing the patient has been presented in the bioethical literature as optimal for such situations [18].

Descriptive statistics were used. To compare means of pain reduction after the application of phenytoin 10% and placebo cream on different areas in the same patient, the Wilcoxon signed-rank test was performed (matched pairs). We calculated the number of patients achieving minimum pain relief (MPR) from baseline of 30% (moderate benefit: MPR30) and 50% (considerable benefit: MPR50) measured on the NRS. The McNemar’s test was used for the dichotomous variables MPR30 and MPR50 to determine statistically significant differences between the areas on which phenytoin 10% and the placebo were applied. As described earlier, the standard phenytoin plasma determination was measured with high-performance liquid chromatography of the plasma protein bound phenytoin concentration, which is approximately 90% of the total phenytoin in plasma [12,19]. The statistical analysis was performed using SPSS 22 (SPSS Inc., Chicago, IL, USA). The presented data are results from clinical observations in our daily practice. The use of the SIBRET did not change the normal clinical procedures leading to prescription of active creams. Furthermore, according to Dutch law on the conduction of medical research, and in line with European guidelines, the response test is not aimed to answer a question in the area of disease and health by systematically collecting and studying data contributing to medical knowledge that also applies to populations outside the direct research population. Therefore, no approval from the ethics committee is required.

## 3. Results

In total, 21 patients were entered into the SIBRET. Nine patients were female (42.9%) and 12 patients were male (57.1%). The age of the patients ranged between 49 years and 89 years, with a mean age of 64.4 years (SD: 9.2). The diagnoses are summarized in Table 1. Most of the patients (N = 12, 57.1%) experienced neuropathic pain only in both feet; other locations are summarized in Table 2. The duration of the neuropathic pain ranged between 1 to 150 months, with a mean duration of 42.2 months (SD: 45.0). More than half of the patients (N = 11, 52.4%) had formerly used neuropathic pain medication (e.g., pregabalin, gabapentin, amitriptyline), though most of them (N = 7, 33.3%) stopped using neuropathic pain medication, either because of no analgesic effect or too many side effects.

The mean reduction on the NRS in the phenytoin 10% area was 3.3 (SD: 1.3) and in the placebo area 1.2 (SD: 1.1) within 30 min. The difference of the mean percentage pain reduction between phenytoin 10% cream and placebo cream was 33.2% (SD: 17.6. A Wilcoxon signed rank test showed that there was a significant difference (Z = −3.9, *p* < 0.001) between scores. In total, 15 patients (71.45%) experienced at least 2-points pain reduction difference on the NRS, in favor of the phenytoin 10% cream. An exact McNemar’s test determined that there was a statistically significant difference in the proportion of MPR50 after application of phenytoin 10% cream and placebo cream, *p* = 0.002 (Table 3). The same holds true for MPR30, *p* < 0.001 (Table 3).

In 6 patients from this cohort of 21 patients, phenytoin plasma levels were determined after 1 to 2 weeks of phenytoin 10% cream application. Plasma sampling was performed 1.5 to 3 h after the last application. No phenytoin plasma levels were detected (below the limit of detection). In 10 other patients, no plasma levels were detected even after the application of 6.7 g of phenytoin 10% cream in one case [12].

## 4. Discussion

In our clinic, we see many patients suffering from localized neuropathic pain. Most patients suffer from pain due to polyneuropathy, which is a symmetrical pain, often burning, that keeps them awake during the night. The fact that in localized neuropathic pain, sensations are often symmetrical, provoked us to develop blind intra-patient test paradigm (SIBRET and DOBRET), in essence these are pharmacological challenge paradigms. All patients suffering from localized neuropathic pain, and with a pain score at least 4 on the NRS, were entered in this test.

Patients had a mean base-line pain score of around 6 on the NRS. The placebo cream resulted in a mean reduction of 1.2 on the NRS. The phenytoin 10% cream resulted in a mean reduction of pain of 3.3 on the NRS, which was more than 50% mean reduction compared to baseline. Placebo cream reduced pain in a much lesser way, and the magnitude of the effect is not considered to be clinically meaningful.

The strength of our SIBRET paradigm is that we can monitor the response of the patient within a short period of time, and the patient is his/her own control. Because no phenytoin plasma levels were found and the pain reduction in responders occurred within 30 min, local pain reducing mechanisms are very likely (e.g., influence at the nerve terminals in stratum granulosum). The judicious use of the placebo in a therapeutic context needs not automatically entail a violation of the doctor’s obligation to heal, depending on the means by which the placebo is presented to the patient [18]. In the case of the SIBRET, the outcome will become clear within 30 min and no further placebo treatment is prescribed. In case a patient reacts positively to the placebo cream, other still unknown mechanisms could be present besides the placebo effect, such as creating a layer on the skin, reducing allodynia, or stimulation of A-beta fibers (touch) reducing pain sensation. Furthermore, a test with placebo and active cream at the same time is important considering that response rates on topical placebos in clinical trials are about twice those seen with oral placebos [20]. Finally, a non-responder to phenytoin cream will receive a prescription for another active cream based on amitriptyline. There is literature on the analgesic effects of topical amitriptyline [21,22,23]. To further define the optimal cut-off value for the SIBRET, a future randomized clinical trial enriched with the double-blind placebo-controlled response test (DOBRET) will performed. The fast onset of action of phenytoin cream (within 30 min), without reaching the blood stream [12], strongly indicates that molecular targets of topical phenytoin are residing in the epidermis. Sodium channels are the main responsible channels for electrogenesis, which is the basis of the action potential generation and its propagation [24]. Neuropathic pain generates a local accumulation of sodium channels at the nociceptors [24,25]. Another molecular mechanism of hyperexitability is cAMP-phosphorylation of the sodium channels [24]. The current knowledge is that sodium channels are involved in the development and maintenance of localized neuropathic pain and it is likely that a general increased excitability, due to the dysfunctional expression of several sodium channels in sensory neurons, leads to the development of neuropathic pain [26]. Therefore, a combined blockade of peripherally expressed isoforms Nav1.7, Nav1.8, and Nav1.9 may prove useful in neuropathic pain [26].

Phenytoin is a broad acting sodium channel blocker [27], and used topically, might be the optimal candidate to block these overactive sodium channels. Phenytoin has low affinities at the resting state of sodium channels, compared to its high affinity at the open/inactivated state of the sodium channel [28]. This explains why no numbness is reported after topical use of phenytoin cream, and can explain the hyperactivity reducing effect in neuropathic pain, acting on a broad spectrum of sodium channels.

## 5. Conclusions

We have noticed that patients are able to distinguish between analgesic effects of different creams when applied to both feet. The special situation of being able to compare within the same time frame, both the effects of active medication and of placebo makes this pharmacological challenge paradigm unique and quite well adapted for testing topical analgesics. 

This SIBRET in 21 patients serves as a proof of principle supporting the pain reducing effect of 10% phenytoin cream in the treatment of localized neuropathic pain.

## Figures and Tables

**Table 1:** Diagnosis.

Diagnosis	N
Idiopathic peripheral neuropathy	6
CIAP	3
CIPN	3
DM type II neuropathy	3
SFN	2
PHN	2
Post Guillain Barre	1
HMSN type 2	1

CIAP: chronic idiopathic axonal polyneuropathy, CIPN: chemotherapy induced polyneuropathy, DM: diabetes mellitus, SFN: small fiber neuropathy, PHN: post-herpetic neuralgia, HMSN: hereditary motor and sensory neuropathy.

**Table 2:** Location of neuropathic pain.

Locations	N
Both feet	12
Both feet and lower legs	6
Complete legs and feet	1
Right upper leg	1
Left belly	1

**Table 3:** Comparisons of effect between phenytoin 10% and placebo cream application.

Characteristics and Effect	Phenytoin 10%	Placebo NRS
Pre-treatment NRS (SD)	6.4 (1.3)	6.2 (1.4)
Post-treatment NRS (SD)	3.1 (1.7)	5.0 (1.6)
Mean pain reduction % (SD)	53.1% (21.2) ^†^	19.9% (17.3)
MPR50 % (N)	57.1% (12) *	9.5% (2)
MPR30 % (N)	85.7% (18) **	23.8% (5)
Mean onset of effect in minutes (SD) (N)	16.3 (9.5) (21)	18.8 (9.7) (13)

^†^*p* < 0.001 with Wilcoxon signed rank test * *p* = 0.002 with the McNemar’s test, ** *p* < 0.001 with the McNemar’s test, NRS: 11-point numerical rating scale, SD: standard deviation, MPR: minimum pain relief.
